# Paratesticular sarcomas: A report of seven cases

**DOI:** 10.3892/ol.2014.2629

**Published:** 2014-10-23

**Authors:** YAŞAR ÜNLÜ, GÜLBEN ERDEM HUQ, GÜLZADE ÖZYALVAÇLI, MEHMET ZENGIN, SEVIM BAYKAL KOCA, UĞUR YÜCETAS, EROL RÜŞTÜ BOZKURT, KEMAL BEHZATOĞLU

**Affiliations:** 1 Department of Pathology, Konya Education and Research Hospital, Konya 42014, Turkey; 2 Department of Pathology, Istanbul Education and Research Hospital, Istanbul 34098, Turkey; 3 Department of Pathology, Faculty of Medicine, Izzet Baysal University, Bolu 14012, Turkey; 4 Department of Pathology, Şırnak Hospital, Şırnak 73002, Turkey; 5 Department of Pathology, Patnos Hospital, Ağrı 04001, Turkey; 6 Department of Urology, Istanbul Education and Research Hospital, Istanbul 34098, Turkey

**Keywords:** paratestis, sarcomas, dedifferentiated liposarcoma, fibromyxois sarcoma, leiomyosarcoma, spindle cell liposarcoma, pleomorphic sarcoma, whorl pattern

## Abstract

Primary tumors of the paratesticular region are rare, with paratesticular sarcomas constituting a major proportion of these tumors, particularly in the elderly. The paratesticular region consists of mesothelial, various epithelial and mesenchymal cells and may therefore give rise to a number of tumors with various behaviors. Defining the association between the paratesticular mass and the testicle, and differentiation between benign and malignant masses using radiology is challenging, therefore the mass is usually considered to be malignant and radical orchiectomy with high ligation is performed. The present study reports the cases of seven patients with tumors of the paratesticular region and presents the clinical and significant histological features of the tumors. In total, two patients suffered from dedifferentiated liposarcoma (DDLS), two exhibited leiomyosarcoma, two exhibited low-grade fibromyxoid sarcoma and one case of undifferentiated pleomorphic sarcoma was identified. Radical orchiectomy with high ligation was performed in five cases; simple orchiectomy was performed in one case and excisional biopsy was performed in the remaining case. A leiomyosarcomatous and epithelial membrane antigen (EMA) positive whorl pattern was observed during microscopy in the two DDLS cases. Additionally, one of the low-grade fibromyxoid sarcoma patients exhibited pleomorphism and mitosis in focal areas. To the best of our knowledge, the present study is the second time low-grade fibromyxoid sarcoma cases with paratesticular localization have been reported in the literature. Of the seven cases, four patients succumbed to the disease, one patient is living with the disorder and the two cases of DDLS are living without the disease. Paratesticular sarcomas are often aggressive and a multidisciplinary approach is required for the diagnosis and treatment of these tumors.

## Introduction

As the paratesticular region contains various structures, including the epididymis, spermatic cord, tunica vaginalis and strong fat-ligament-muscle supporting tissues, it may give rise to a number tumor types with various behaviors (1). Tumor variability may also be due to the Wolffian duct origin of the testis appendages, including the spermatic cord. The most significant feature of the paratesticular region is that it is the origin of a small number of tumors with rich diversity.

The majority of the masses within the scrotum in adults are of testicular origin. Paratesticular masses account for 2–3% and sarcomas account for ~30% of all scrotal masses (1–4). The most common type of sarcoma is liposarcoma, followed by leiomyosarcoma (LMS), rhabdomyosarcoma (RMS), undifferentiated pleomorphic sarcoma and fibrosarcoma (1–6). To the best of our knowledge, only one instance of low-grade fibromyxoid sarcoma in a paratesticular location has been previously reported (7).

Determining the association between the paratesticular mass and the testicle, and differentiation between benign and malignant masses using radiology is challenging, therefore the lesions are usually considered to be malignant and radical orchiectomy with high ligation is used. The prognosis is often poor, as recurrence and metastasis are common, and the mechanism and outcome of regional lymph node resection, radiotherapy and chemotherapy is unclear. The present study reports seven cases of paratesticular sarcoma and emphasizes the significant clinical and histological features.

## Case reports

Seven cases of paratesticular sarcoma diagnosed at the Pathology Department of the Istanbul Education and Research Hospital (Istanbul, Turkey) are retrospectively investigated. Hematoxylin and eosin and immunohistochemical staining of the cases were reevaluated and accurately diagnosed according to the recent World Health Organization classification (8). The clinical information of the patients was obtained from the patient files. Written informed consent was obtained from all patients. All patients had been referred to the Urology clinic at Istanbul Education and Research Hospital with a growing scrotal mass. Excisional biopsy and simple orchiectomy were performed in cases three and four and the two patients were subsequently diagnosed with fibromyxoid sarcoma. Radical orchiectomy was performed in the other five cases. Cases one, two and three did not receive any additional treatment, whereas chemotherapy and radiotherapy was adminstered to case five. In cases four, six and seven, re-excision was performed due to recurrence, and chemotherapy and radiotherapy were adminsitered following re-excision.

**Figure 1:**
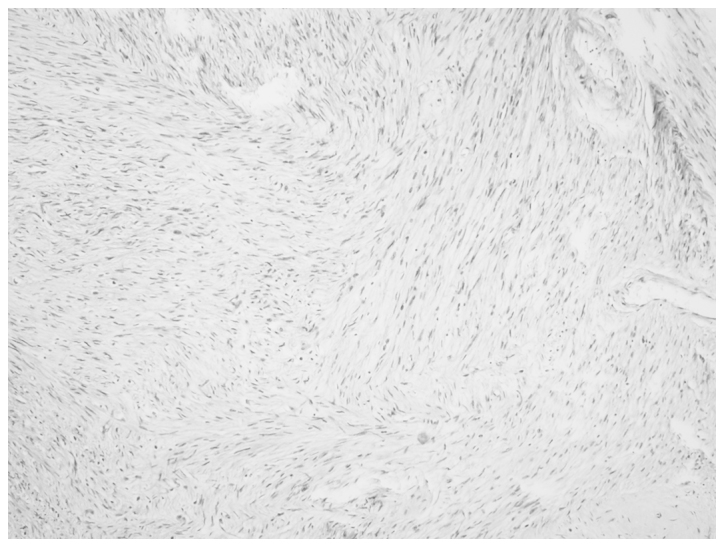
Hematoxylin and eosin staining revealing well-differentiated smooth muscle with a low nuclear grade in dedifferentiated liposarcoma (magnification, ×200).

**Figure 2:**
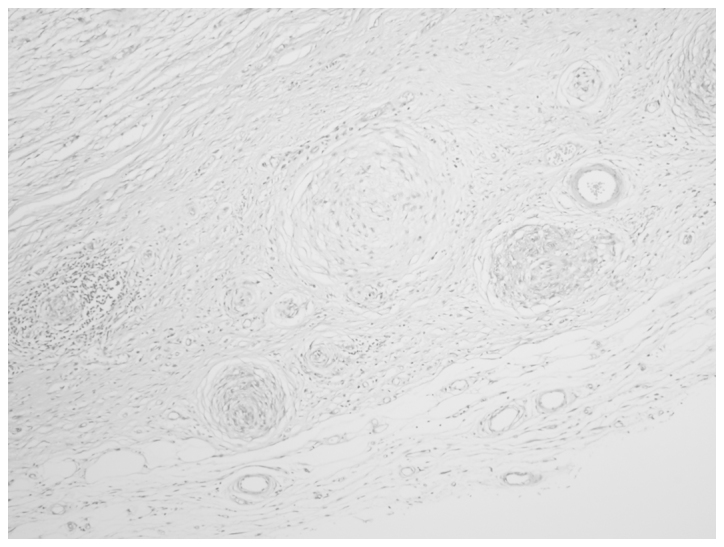
Hematoxylin and eosin staining revealing a whorl pattern in dedifferentiated liposarcoma (magnification, ×100).

**Figure 3:**
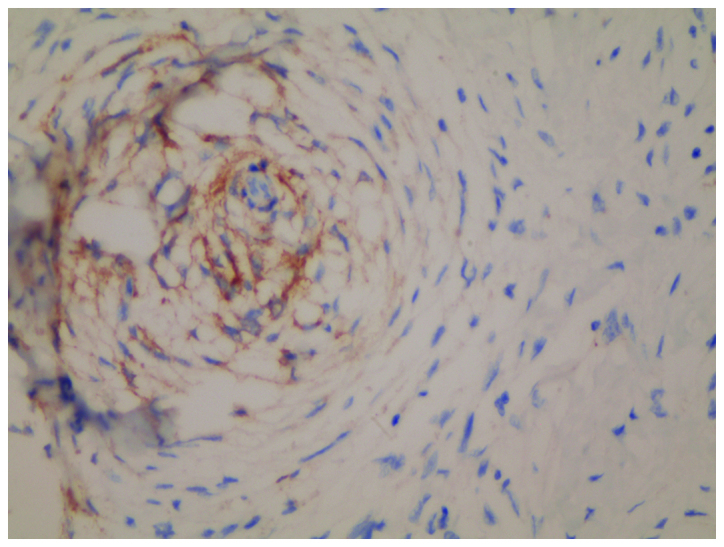
Immunohistochemical staining was positive for epithelial membrane antigen (magnification, ×400).

**Figure 4:**
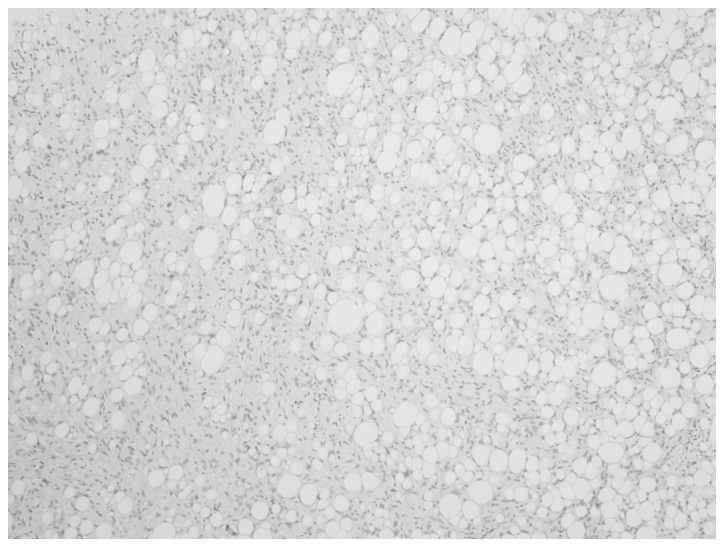
Hematoxylin and eosin staining revealing the spindle cell liposarcoma area in dedifferentiated liposarcoma (magnification, 100).

**Figure 5:**
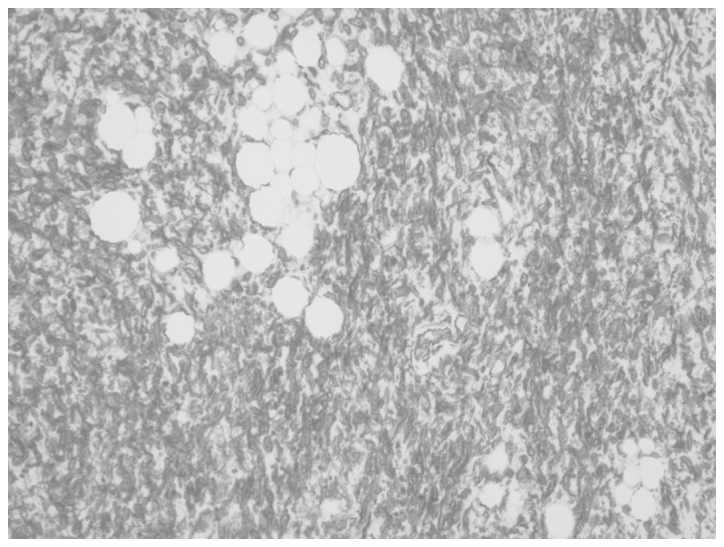
Immunohistochemical staining of the spindle cell liposarcoma area in dedifferentiated liposarcoma was positive for CD34 (magnification, ×300).

The clinical features are summarized in Table I. The macroscopic and histological characteristics of the cases were as follows: The lesions in cases one and two consisted of large yellow (lipomatous) and well-delineated areas, with occasional tan-gray colored (leiomyosarcomatous) areas. The diameter of the leiomyosarcomatous area was 8 cm in case one and 5 cm in case two. In addition, a few gray-colored nodules, the largest with a 1 cm diameter, were present in case two. A homologous pattern consisting of well-differentiated liposarcoma, comprising predominantly spindle cells, was present in each case (Fig. 1). The heterologous pattern consisted of a LMS and meningothelial-like whorl component in each case and a low-grade chondrosarcoma component in case one was also present (Figs. 2 and 3). Immunohistochemical staining of the cells revealed that the whorl pattern area was positive for epithelial membrane antigen (EMA; Fig. 4). A strong positive result for cluster of differentiation (CD)34 was observed in the spindle cells within the spindle cell liposarcoma areas, regarded as the homologous component (Fig. 5). The two cases were diagnosed as leiomyosarcomatosis and dedifferentiated liposarcoma (DDLS) containing whorl-pattern areas.

**Figure 6:**
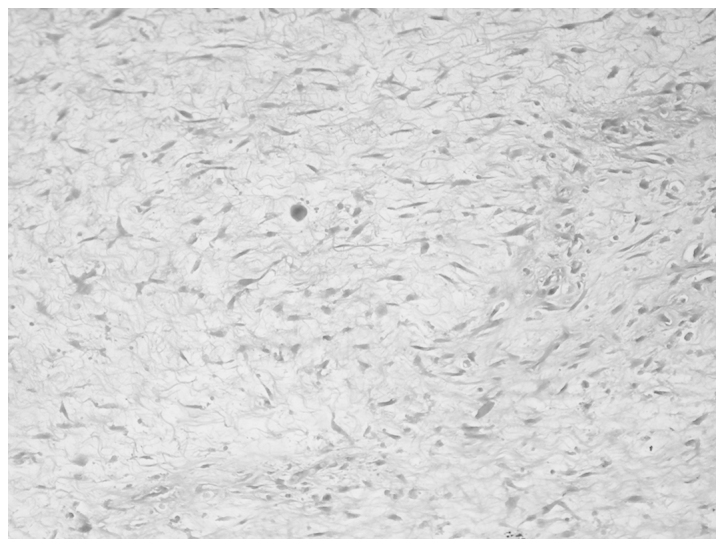
Hematoxylin and eosin staining of low-grade fibromyxoid sarcoma, revealing bland fibroblasts with myxoid stroma and a rich capillary network (magnification, ×200).

**Figure 7:**
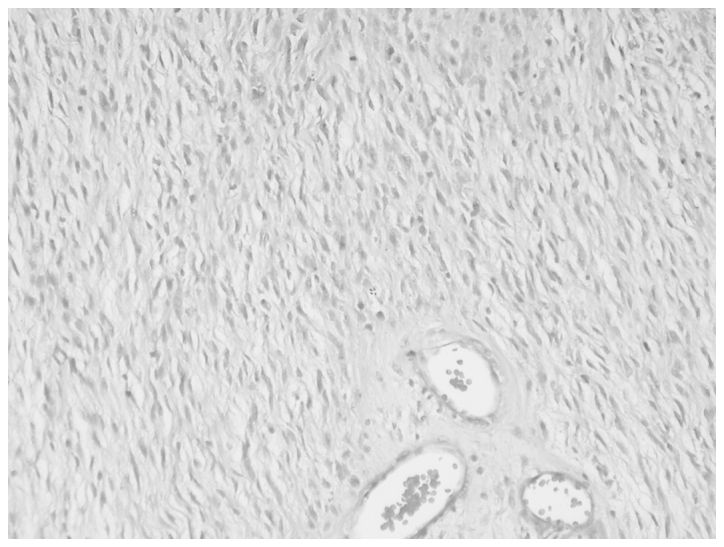
Hematoxylin and eosin staining of low-grade fibromyxoid sarcoma, revealing thick-walled vessels and bland fibroblasts (magnification, ×300).

**Figure 8:**
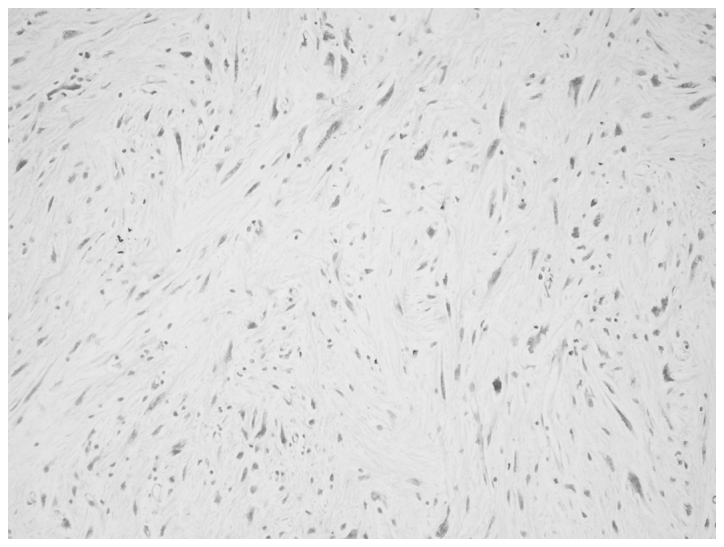
Hematoxylin and eosin staining of low-grade fibromyxoid sarcoma, revealing nuclear pleomorphism (magnification, ×300).

Cases three and four exhibited nodular, well-delineated tumors with surgical border invasion. Microscopy revealed the histology to be similar in the two cases, with the prominent features consisting of a collagen and myxoid zone, mixed with bland spindle-like fibroblastic cells and a whorl pattern, and arcades of curvilinear blood vessels (Figs. 6 and 7). The cellularity of the tumors varied from extremely low to moderate. Pleomorphism and mitosis were present in the nuclei in a focal area in case four (Fig. 8). Immunohistochemical staining for vimentin and MUC4 yielded a positive result in the two cases, together with focal CD34 and EMA positivity. The patients were diagnosed with low-grade fibomyxoid sarcoma.

The masses in cases five and six were well-circumscribed and nodular, with long bundles that were parallel or perpendicular to each other. The cell cytoplasm was strongly eosinophilic and the nuclei were generally spindle shaped, with one blunt end. Mitosis was not frequent, with 1–3 mitoses per 10 high power fields. Necrosis was observed in focal areas and pleomorphism was moderate. Immunohistochemical staining for smooth muscle actin and desmin yielded a positive result. The two cases were diagnosed as LMS.

Macroscopically, the mass in case seven had infiltrative borders, and, microscopically, was rich in spindle cells, with small bundles and storiform patterns. The tissue also contained large pleomorphic cells, with large eosinophilic cytoplasm in certain areas. Immunohistochemical staining yielded a positive result for vimentin only. This case was diagnosed as undifferentiated pleomorphic sarcoma.

## Discussion

Paratesticular sarcomas are rare and account for ~2% of all soft tissue sarcomas (9,10). No clear approach is available regarding their behavior and treatment due to their rarity. Liposarcoma and LMS are the most common sarcomas (1–6). RMS is more frequent in younger patients (6). The development of various paratesticular neoplasia is due to the differing complex structures in the region. In addition, embryological development of the spermatic cord, the most common tumor localization, and the other testicular adnexal structures from the Wolffian duct may be responsible for this histological diversity.

The histological features of the three cases diagnosed with LMS and undifferentiated pleomorphic sarcoma were typical and created no diagnostic problems. However, the two DDLS cases exhibited extremely rare histological features.

Although the LMS and whorl patterns observed in the DDLS cases is not common, they have been described in previous studies (11–13). Leiomyosarcomatous areas were observed in each of the DDLS cases. The cases exhibited low-grade morphology and extremely low rates of mitosis, with mild pleomorphism in focal areas. The positive immunohistochemical staining for EMA in the whorl pattern areas was noteworthy and suggested perineural or meningeal differentiation. Meningothelial whorl-like morphology is less frequently observed than LMS. EMA was present in each case and, to the best of our knowledge, these are the first DDLS cases positive for EMA to be reported.

A further feature of the DDLS cases was the dominant spindle cell liposarcoma as a homologous component. The denomination of spindle cell liposarcoma remains controversial; certain authors use spindle-cell neoplasm or spindle cell lipoma-like neoplasm (14), and the recommended name for certain lesions was fibrosarcoma-like lipoid neoplasm, as determined in a report published by Deylup *et al* (15). The authors who recommend these names do not classify these tumors as liposarcoma, however, lipoblasts were observed. The two DDLS cases in the present study were rich in spindle cell lipoma-like (spindle cell liposarcoma) areas with strong cytoplasmic CD34-positivity, as well as rich in lipoblasts. These areas, which were the homologous component for DDLS, should be classified as spindle cell liposarcoma.

Low-grade fibromyxoid sarcoma is a relatively rare sarcoma (16–17). The upper extremities and the torso are the most frequent locations, however, no paratesticular localization has been previously reported. Low-grade fibromyxoid sarcoma usually exhibits variable microscopic findings, with bland fibroblasts, whorls, linear sequencing and less cellular myxoid sections in certain areas (16). Mitosis and necrosis are rare. One of the present cases exhibited typical features of low-grade fibromyxoid sarcoma, while case four exhibited pleomorphism in focal areas that were also rich in mitoses.

The accepted treatment for paratesticular masses is radical inguinal orchiectomy, including the surrounding soft tissues. No consensus with regard to regional lymph node excision has been reached, radiotherapy and chemotherapy. In the current study, radical orchiectomy with high ligation was performed for the seven cases. Simple orchiectomy and excisional biopsy were conducted for the two cases with fibromyxoid sarcoma. The two patients with fibromyxoid sarcoma succumbed to the disease within 22 and 43 months, respectively, as there was residual mass and the patients did not accept additional treatment. This emphasizes the importance of radical surgical treatments. No recurrence or metastasis was observed in the two cases of liposarcoma and the improved prognosis of liposarcoma compared with the other sarcomas, or the short clinical follow-up durations may have had an effect on this finding. As four of the seven cases succumbed to the disease and one remains alive with the sarcoma demonstrates the requirement for a multidisciplinary approach to the treatment of paratesticular sarcomas.

In conclusion, the paratesticular region consists of complex structures that can develop various neoplastic formations and patterns. Sarcomas comprise a significant part of paratesticular masses and may exhibit an aggressive clinical course. In older patients, paratesticular sarcomas must be considered for the differential diagnosis of scrotal masses, which do not exhibit a clear association with the testes. Furthermore, clinicians and patients must be informed about the high probability of local recurrence and distant metastasis in paratesticular sarcomas.

## Figures and Tables

**Table I:** Clinical findings and diagnosis of paratesticular sarcoma.

Case	Age, years	Diagnosis	Tumor size, cm	Treatment	Additional treatment	Follow-up time, months	Disease outcome
1	70	Dedifferentiated liposarcoma	13.0	Radical orchiectomy with high cord ligation	None	14	Survival
2	38	Dedifferentiated liposarcoma	13.0	Radical orchiectomy with high cord ligation	None	8	Survival
3	72	Fibromyxoid sarcoma	7.0	Excisional biopsy	None	22	Mortality
4	63	Fibromyxoid sarcoma	9.0	Simple orchiectomy	Re-resection, CTh + RTh	43	Mortality, recurrence with lung metastasis
5	64	Leiomyosarcoma	6.5	Radical orchiectomy with high cord ligation	CTh + RTh	21	Mortality, recurrence with lung metastasis
6	68	Leiomyosarcoma	7.4	Radical orchiectomy with high cord ligation	Re-resection, CTh + RTh	18	Survival, recurrence
7	46	Undifferentiated pleomorphic sarcoma	4.2	Radical orchiectomy with high cord ligation	Re-resection, CTh + RTh	44	Mortality, recurrence

CTh, chemotherapy; RTh, radiotherapy.
